# Comparative Study on Physicochemical and Nutritional Qualities of Kiwifruit Varieties

**DOI:** 10.3390/foods12010108

**Published:** 2022-12-25

**Authors:** Xinyu Yuan, Hao Zheng, Jiangtao Fan, Fengxia Liu, Jitao Li, Caihong Zhong, Qiong Zhang

**Affiliations:** 1 Key Laboratory of Plant Germplasm Enhancement and Specialty Agriculture, Wuhan Botanical Garden, Chinese Academy of Sciences, Wuhan 430074, China; 2 School of Forestry and Horticulture, Hubei Minzu University, Enshi 445000, China; 3 College of Food Science and Technology, Huazhong Agricultural University, Wuhan 430070, China

**Keywords:** nutritional quality, kiwifruit, species, flesh color, principal component analysis

## Abstract

In order to study the physicochemical and nutritional characteristics of kiwifruit varieties, 14 kiwifruits from different species with different flesh colors were selected for research. The pectin content was significantly higher in green-fleshed kiwifruits than those in red-fleshed and yellow-fleshed kiwifruits. Red-fleshed kiwifruits had the highest total flavonoid content, and green-fleshed kiwifruits in *A. eriantha* had the highest chlorophyll a content, chlorophyll b content and total carotenoid content. The energy and carbohydrate contents of yellow-fleshed kiwifruits were significantly lower than those of red-fleshed kiwifruit. Moreover, the protein contents in *A. chinensis* and *A. chinensis* var. *deliciosa* were higher than those in other species. The content of vitamin C in *A. eriantha* was far higher than in other kiwifruits. Red-fleshed kiwifruits had a significantly higher vitamin E and vitamin B1 content than green-fleshed kiwifruits. In addition, 1-pentanol, trans-2-hexen-1-ol, n-hexane and styrene presented only in red-fleshed kiwifruits. Therefore, these could be used as a characteristic fragrance for red-fleshed kiwifruits. Moreover, the varieties were ranked comprehensively by principal component analysis (PCA), among which the top four highest-ranking kiwifruits among the 14 varieties were ‘Huate’, ‘MHYX’, ‘Jinkui’ and ‘Xuxiang’, respectively. This study provides a reference for consumers and markets on quality improvement and processing.

## 1. Introduction

The kiwifruit is a perennial deciduous vine that belongs to the genus Actinidia. The genus Actinidia has 75 taxa, comprising 54 species and 21 variations, as of the most recent classification [1]. China is endowed with a wealth of genetic resources because it is the origin area of kiwifruit. Currently, the commercial kiwifruit cultivars are mainly from *A. chinensis* and *A. chinensis* var. *deliciosa*, and a small amount of *A. eriantha* and *A. arguta*. According to the fruit flesh color of kiwifruits, they are mainly divided into three categories: green-fleshed, yellow-fleshed and red-fleshed. At present, more than 90% of cultivated kiwifruits are green and yellow-fleshed varieties. Green-fleshed ‘Hayward’ is the main plant variety around the world, and is loved by consumers because of its strong aroma, excellent quality, good storage and other outstanding advantages [2]. Yellow-fleshed ‘Jinyan’ is tender, juicy and fragrant, with extremely disease-resistant fruit, which is recognized by domestic and international markets [3]. In recent years, due to their unique flavor and nutritional value, red-fleshed kiwifruits have become increasingly popular with consumers [4,5]. ‘Donghong’ is a new variety of red-fleshed kiwifruit with strong disease resistance, heat and drought tolerance, brilliant red inner skin and sweet flavor [6]. 

As we know, kiwifruits with high nutritional quality, good flavor and aromatic scent [7] have important commercial value [8]. In recent years, the demand for high fruit quality has increased gradually, and studies have shown that fruit nutritional value is critical to fruit quality, which will provide consumers with a basis for consumption [9]. However, fewer studies have been conducted on the nutritional variation among different varieties, and some popular varieties bred in recent years have not been studied [10].

Therefore, in order to explore the profile of nutritional variation in each kiwifruit variety, we selected 14 kiwifruit varieties, covering red-fleshed, green-fleshed and yellow-fleshed kiwifruits, which are widely cultivated or have high market shares in China. In this study, the pectin, moisture, chlorophyll, carotenoid, flavonoid, energy, carbohydrate, protein, lipid, dietary fiber, soluble sugar, organic acid, vitamin and aroma compound contents were detected and compared. The physical and chemical properties, nutritional properties and aroma of various kiwifruit varieties provide a reference for their consumption and utilization, as well as for kiwifruit breeding.

## 2. Materials and Methods

### 2.1. Materials and Reagent

In this study, ‘Hort16A’ and ‘G3′ were sourced from supermarkets, and the other 12 kiwifruit cultivars were obtained from the base of the National Germplasm Repository of Kiwifruit in Hubei Province in 2021. They were divided into three main categories: red-fleshed kiwifruits (R)—‘Donghong’ and ‘Hongyang’; green-fleshed kiwifruits (G)—‘Jinkui’, ‘Cuiyu’, ‘Xuxiang’, ‘Hayward’, ‘MHYX’ and ‘Huate’; and yellow-fleshed kiwifruits (Y)—‘Hort16A’, ‘G3’, ‘Jintao’, ‘Jinyuan’, ‘Jinmei’ and ‘Jinyan’ in Table 1. Among all 14 kiwifruit cultivars, 6 were from *A. chinensis*, 3 were from *A. chinensis* var. *deliciosa*, 2 were form *A. eriantha*, and 3 were interspecific hybrids of *A. eriantha* and *A. chinensis*. The cessation of fruit growth (size and weight), closely matched to the maximum of dry matter, with 100% of black seed are considered to be the first sign for kiwifruit to be mature [11]. The kiwifruit trees began to bear fruit more than 5 years ago and fruits were randomly picked at the mature stage with a maximum of dry matter. From each of the 14 groups, a replicate consisted of 10 fruits, and each group had 3 biological replicates. Thirty fruits were used, and all organic solvents were from the China National Pharmaceutical Group.

### 2.2. Methods

#### 2.2.1. Determinations of Pectin and Moisture Content

Determination of pectin content was as referred to in the literature [12] with some modifications, pectin content was measured by the carbazole colorimetric method using galacturonic acid as the standard. In brief, 0.25 mL carbazole-ethanol solution and 6 mL sulfuric acid (98%, *w*/*w*) were successively added into 1 mL pectin solution. The mixture was heated at 85 °C for 30 min, and then cooled to room temperature and kept in the dark for 30 min. The absorbance was measured at 530 nm using a spectrophotometer. Three replicates were performed.

The method of moisture measurement referred to CNS GB 5009.3-2016 by Direct-drying method. A total of 3 g of kiwifruit pulp was placed in a constant weighted weighing bottle and placed in a 100 °C oven for 2 h, chilled in a desiccator for 10 min, then the drying process was repeated until it reached a constant weight.

#### 2.2.2. Determinations of Chlorophyll a Content (CAC), Chlorophyll B Content (CBC), Total Carotenoid Content (TCC)

Measurement of CAC, CBC and TCC was referred to in the literature [13], with some modifications. The absorbances at 470 nm, 649 nm and 665 nm were recorded using a UV spectrophotometer in the range of 350~700 nm and the chlorophyll a, b and total carotenoid contents were calculated according to the following equations.

(1) $$C_{a}\left(\mu g/g\right)=\frac{(13.95A_{665}-6.88A_{649})\times V\times f}{m}$$

(2) $$C_{b}\left(\mu g/g\right)=\frac{\left(24.96A_{649}-7.32A_{665}\right)\times V\times f}{m}$$

(3) $$C_{xc}\left(\mu g/g\right)=\frac{\left(4.08A_{470}+3.31A_{665}-11.64A_{649}\right)\times V\times f}{m}$$

where *C_a_*, *C_b_* and *C_xc_* were the content of chlorophyll a, b and carotenoids (μg/g), respectively. *A*_470_, *A*_649_ and *A*_665_ were the absorbance at 470 nm, 649 nm and 665 nm, respectively. 

#### 2.2.3. Determination of Total Flavonoid Content (TFC)

Measurement of TFC as referred to in the literature [14], with some modifications. The kiwifruit pulp was placed in a drying oven at 55 °C for 6 h, and then weighed and ethanol added at a certain ratio, and then enzymatically digested. After that, the mixture was centrifuged at 4000 r/min for 10 min. The supernatant was filtered to obtain the crude extract of flavonoids. The 3 mL crude extract was then transferred to 10 mL colorimetric tubes and the absorbance A was measured at 510 nm. The mass concentration *C*_0_ of the flavonoids was calculated according to the linear regression equation of the standard curve, and then the total flavonoid content in kiwifruit was calculated according to equation:

(4) $$G\left(\mathrm{mg}/100\;g\right)=\frac{C_{0}\times V_{0}\times N}{W}$$

where *G* was total flavonoid content (mg/100 g), *C*_0_ was mass of flavonoids concentration (mg/mL), *V*_0_ was total volume of sample extracts (mL), *N* was the dilution multiplier of sample extracts, *W* was the weight of samples (g).

#### 2.2.4. Determinations of Energy, Carbohydrates, Protein, Lipid and Dietary Fiber

Both measurement of energy and carbohydrates contents were based on CNS GB 21922. The protein content was determined using a Kjeldahl method with a Kjeltec 2300 Auto Analyzer (FOSS, Hillerød, Denmark) based on CNS GB 5009.5-2010. Measurement of lipid content was based on CNS GB 5009.6-2016 by Soxhlet extractor method. Measurement of dietary fiber content was based on CNS GB 5009.88-2014. 

#### 2.2.5. Determinations of Total Sugar and Total Acid Content

Determination of total sugar content as referred to in the literature [15] for the determination of reducing sugars in foods by anthrone colorimetric method. Each sample was divided into 3 tubes. A total of 2 g of pulp was weighed with a straw and placed in a 50 mL centrifuge tube. Add water about 10 mL first, then slowly add 1 mL zinc acetate solution and 1 mL potassium ferrocyanide solution, shake gently, then add water to the scale, mix upside down, and let it rest for 30 min. After centrifugation at 4000 rpm × 10 min at room temperature, 1 mL of the supernatant was transferred to a 15 mL centrifuge tube, and 9 mL of water was added to obtain a 10-fold diluted sample supernatant. A total of 0.5 mL of the above 10-fold diluted sample supernatant was aspirated and added to a 15 mL centrifuge tube with 1.5 mL of water to determine the absorbance value of the reaction solution at 620 nm. Total acid content as referred to in the literature [9], with some modifications. The 0.1 M sodium hydroxide solution was used to titrate the sample (50 mL) to a pH of 8.1, and the following formula was used to determine the total acid content:

(5) $$TAC\left(g/100\;g\right)=C\times V_{2}\times K/V_{1}\times \left(V_{0}/W\right)\times 100$$

where *TAC* was total acid content (g/100 g), *C* was the sodium hydroxide solution concentration (0.1 M), *W* was the sample mass (g), *V*_2_ was the volume of sodium hydroxide solution consumed by titration (mL), *V*_1_ was the titrated sample volume (mL), *V*_0_ was the total volume of crude extract (mL), *K* was the citric acid conversion coefficient, 0.070.

#### 2.2.6. Determinations of Soluble Sugar and Organic Acid Content

Determination of soluble sugar (sucrose content + glucose content + fructose content) as referred to in the literature [16]. The kiwifruit samples were ground into a fine powder in liquid nitrogen and 1 g of powder was dissolved in 6 mL deionized water that was generated by a Milli-Q Element water purification system (Millipore, Bed ford, MA, USA). The mixture was centrifuged at 5000× *g* for 15 min at 4 °C and the supernatants were filtered through a SPE-C18 cartridge (Supelclean ENVI C18 SPE) and a 0.22 μm millipore membrane. A Dionex P680 HPLC system was used to measure the amount of soluble sugar in the filtered solution (Dionex Corporation, Sunnyvale, CA, USA). The amount of fructose, glucose and sucrose contents were determined using the refractive index detector. The mobile phase was degassed, distilled and deionized water flowing at a rate of 0.6 mL/min. The total injection volume was 20 μL. Samples were eluted with 0.02 mol/L KH_2_PO_4_ solution (pH 2.4). The flow rate was 0.8 mL/min. Eluted compounds were detected by UV absorbance at 210 nm.

Determination of organic acid (malic acid content + citric acid content + tartaric acid content + quinic acid content) as referred to in the literature [17]. A 50 mL centrifuge tube containing 20 g of kiwifruit pulp was then filled with 25 mL of NH_4_H_2_PO_4_ (40 mmol/L, pH 2.5 was adjusted using H_3_PO_4_), followed by 15 min of centrifugation at 12,000× *g* at 4 °C. The supernatants were collected and stored at 4 °C for further analysis. A total of 8 organic acid standards were detected, including oxalic acid, tartaric acid, quinic acid, isocitric acid, malic acid, shikimic acid, lactic acid and citric acid. A Waters Alliance 2695 HPLC system with a 2996 PDA Detector (Waters Corp., Milford, MA, USA) was used to simultaneously separate and analyze the organic acids. The system was run at 1.0 mL/min using a ST-C18 column (250 mm × 4.6 mm, 5 μm) (TechMate, Beijing, China) and a guard column RP-18 (10 mm × 4 mm) (Merck, Darmstadt, Germany). The column temperature was set at 25 °C, and 10 μL of sample was injected. The detection wavelength was 210 nm. The mobile phase was NH_4_H_2_PO_4_ (40 mmol/L, pH 2.5 adjusted using H_3_PO_4_).

#### 2.2.7. Determinations of Vitamin C (VC), Vitamin B1 (VB1), Vitamin B2 (VB2), Vitamin B6 (VB6) and Vitamin E (VE)

The VC content was determined using a 2,6-dichloroindophenol titration method based on CNS GB/T 6195-1986. Put 100 g kiwifruit fruits into a masher, add 100 mL extract, and quickly mash them into a homogenate. Then 20 g of the slurry sample was weighed, the sample was transferred to a 100 mL volumetric flask with extractant, and dilute to a certain extent, and then shaken and filtered. If the filtrate is colored, it can be decolorized by adding 0.4 g porcellanite per gram sample and then filtered. A total of 10 mL of filtrate was aspirated into a 50 mL conical flask with calibrated 2,6-dichloroindophenol solution and was titrated until the solution was pink and did not fade for 15 s. A blank test was performed at the same time. Measurement of VB1 content referred to CNS GB 5009.84-2016. Measurement of VB2 content referred to CNS GB 5009.85-2016. Measurement of VB6 content referred to CNS GB 5009.154-2016. Measurement of VE content referred to CNS GB 5009.82-2016. 

#### 2.2.8. Determination of Aroma

Determination of aroma substance as referred to in the literature [18], with minor modifications. A total of 2.0 g of pulverized kiwifruit tissue and 0.6 g sodium chloride were placed into a headspace sample vial (20 mL) containing a magnetic rotor. After the sample vial was sealed with a PTFE/silicone septum and an aluminum cap, it was placed into a water bath at 40 °C with magnetic stirring that the rotor can rotate in the fruit tissue. Meanwhile, a manual SPME device with holder and embedded fiber pierced through the septum and reached the headspace above the sample. After the fiber was stretched out and exposed to headspace volatiles for 40 min, it was then withdrawn and the whole holder was transferred to the GC injection port for desorption for 10 min at a temperature of 250 °C. In the meantime, the GC–MS system was triggered for the separation and detection of the desorbed volatiles.

The SPME was carried out using a manual device with holder and fiber from Supelco (Belefonte, PA, USA). The fiber used for extracting volatiles is divinlbenzene/carboxen/poly(dimethylsiloxane) (DVB/CAR/PDMS). The GC–MS analysis of the volatile compounds from kiwifruit was employed using a GC system (Agilent 7890A, Palo Alto, CA, USA) equipped with HP-5 capillary column, and MS system (Agilent 7000C, Palo Alto, CA, USA). GC separation of the volatiles was conducted under carrier gas of helium, which was at a flow rate of 1 mL/min. The column temperature program of GC was initially set at 40 °C for 1 min, and gradually increased to 230 °C at 15 °C/min, then kept there for 5 min. For GC–MS detection, electron ionization (EI) system for acquiring mass spectra were over the mass range of 35–350 amu, and the ionization energy used was at 70 eV.

### 2.3. Statistical Analysis

The mean and standard deviation of three parallel measurements were used to express the experimental results. SPSS 26 and RStudio were used to conduct the statistical analysis. The graphing was performed using GraphPad Prism9.

## 3. Results

### 3.1. Pectin and Moisture

**Figure 1:**
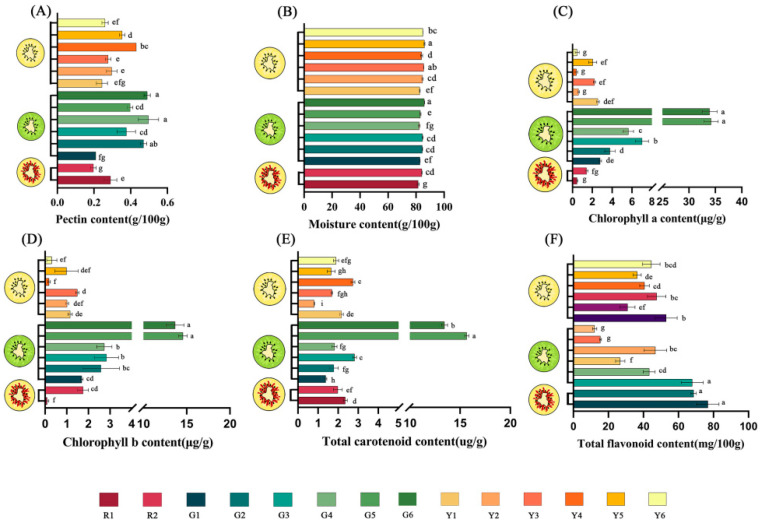
The contents of pectin, moisture, chlorophyll a, chlorophyll b, total carotenoids and total flavonoids. (**A**) The contents of pectin. (**B**) The contents of moisture. (**C**) The contents of chlorophyll a. (**D**) The contents of chlorophyll b. (**E**) The contents of total carotenoids. (**F**) The contents of total flavonoids. ‘R1’, ‘Hongyang’. ‘R2’, ‘Donghong’. ‘G1’, ‘Cuiyu’. ‘G2’, ‘Xuxiang’. ‘G3’, ‘Hayward’. ‘G4’, ‘Jinkui’. ‘G5’, ‘MHYX’. ‘G6’, ‘Huate’. ‘Y1’, ‘Hort16A’. ‘Y2’, ‘G3’. ‘Y3’, ‘Jintao’. ‘Y4’, ‘Jinyuan’. ‘Y5’, ‘Jinmei’. ‘Y6’, ‘Jinyan’. Different lowercase letters indicated statistically significant differences (Duncan’s multiple range test, *p* < 0.05) among different kiwifruit cultivars.

In the case depicted in Figure 1A, the pectin contents of kiwifruits ranged from 0.20 to 0.50 g/100 g, with an average of 0.34 g/100 g. Among the kiwifruits with different fruit flesh colors, the green-fleshed kiwifruits showed the highest content of pectin compared with the red-fleshed and yellow-fleshed kiwifruits, and this case is depicted in Figure S1A. On the other hand, ‘Donghong’ showed the lowest content of pectin, followed by ‘Cuiyu’ and was ‘Hort16A’—these three varieties belonged to *A. chinensis*. Except for *A. chinensis* var. *deliciosa* vs. *A. eriantha*, the content of pectin in each different species was significantly different. It was reported that the pectin content of green-fleshed ‘Qinmei’ kiwifruit, which was from *A. chinensis* var. *deliciosa*, ranged from 0.09 g/100 g to 0.37 g/100 g [19]. In this study, the pectin contents of five green-fleshed kiwifruit samples from *A. chinensis* var. *deliciosa* and *A. eriantha* were higher than those in a previous study. The green-fleshed kiwifruits with a high pectin content in *A. chinensis* var. *deliciosa* and *A. eriantha* are a natural source of pectin, which could be used in the preparation of jellies and jams. 

The content of moisture plays critical roles in the shelf life and processing characteristics of the fruit. The results demonstrate that there were significant differences in the moisture content of the various samples, which spanned from 81.49% to 85.96%, and ‘Jinmei’ showed the highest content of moisture. Based on the flesh color, no significant difference was identified in the moisture contents of the three flesh color groups. Coincidentally, the content of moisture in different species showed no significance, and this case is depicted in Figure S1. It was reported that the moisture content of *A. chinensis* and *A. chinensis* var. *deliciosa* was 83.22% and 83.07%, respectively [20], which was similar to the results of this study. In addition, studies showed that the moisture content in dragon fruit [21], apple [22] and citrus [23] were 82.40–84.80%, 81.66–3.40% and 75.40–80.32%, respectively. Therefore, it can be deduced that the kiwifruits had a high moisture content and are a good supplier of moisture for humans.

### 3.2. CAC, CBC, TCC and TFC

As seen in Figure 1C–F, the results demonstrated that ‘MHYX’ showed the highest CAC (34.18 μg/g), CBC (14.57 μg/g) and TCC (15.66 μg/g); however, it did not exhibit this pattern in the TFC. Fruits of *A. eriantha* with green flesh color had the highest chlorophyll a content and chlorophyll b content. Carotenoids present in kiwifruits contribute to the yellow color of the fruit. Interestingly, the total carotenoid content of *A. eriantha* was also the highest in all species. The CAC, CBC and TCC of green-fleshed kiwifruits was significantly higher than those of yellow-fleshed kiwifruits, as seen in Figure S2. However, the TFC did not show the above pattern. The TFC of red-fleshed kiwifruits was significantly higher than that of green-fleshed and yellow-fleshed kiwifruits. Interestingly, based on the species of the 14 varieties of kiwifruit, TFC exhibited patterns that differ from CAC, CBC and TCC. In summary, *A. eriantha* had significantly higher CAC, CBC and TCC than other samples, whereas *A. chinensis* was significantly higher than other samples in TFC in Figure S2. The reason for this might be that red-fleshed kiwifruits belonged to *A. chinensis*, and the red-fleshed kiwifruits contained more anthocyanins, resulting in a higher content of flavonoids. In addition, ‘Cuiyu’ (*A. chinensis*) had the highest TFC among the green-fleshed kiwifruits.

In kiwifruits, chlorophyll contents were also studied in Jiangxi in previous reports [13,24], and the CAC and CBC for all cultivars in those studies were higher than in this study. Light, temperature, and moisture all affect chlorophyll content [25,26,27], and the reason might lie in the diverse regions, with the average annual temperature in Hubei being much higher than that in Jiangxi. In addition, there were many factors that can affect TCC, such as variety, light and geographic factors [28]. The TCC and TFC detected in *A. chinensis* from the previous results were 0.80 μg/g–3.50 μg/g and 20.80 mg/100 g–66.70 mg/100 g [29], respectively, being similar to this study. Studies have shown that flavonoids can prevent cancer, coronary heart disease, stroke and other diseases as well as having anti-aging effects [1]. Therefore, kiwifruits in *A. chinensis* had the highest TFC, so they are more suitable as a supplementary source of human flavonoids. In addition, green-fleshed kiwifruits in *A. eriantha* had the highest CAC, CBC and TCC.

### 3.3. Energy, Carbohydrates, Protein, Lipid and Dietary Fiber

**Figure 2:**
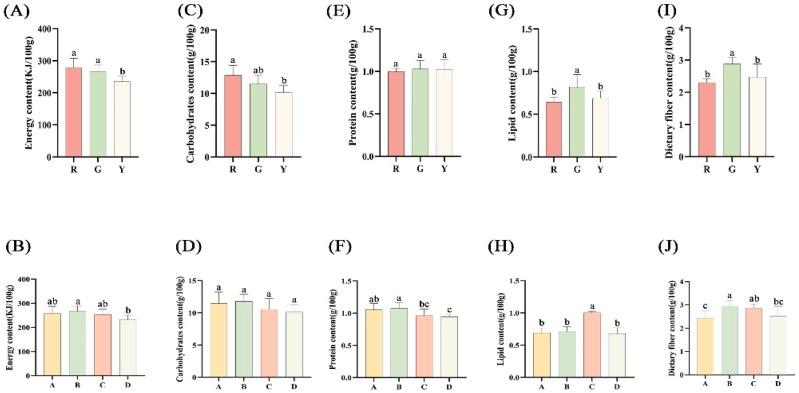
The contents of energy, carbohydrates, protein, lipid and dietary fiber. (**A**) The contents of energy in different flesh color. (**B**) The contents of energy in species. (**C**) The contents of carbohydrates in different flesh color. (**D**) The contents of carbohydrates in species. (**E**) The contents of protein in different flesh color. (**F**) The contents of protein in species. (**G**) The contents of lipid in different flesh color. (**H**) The contents of lipid in species. (**I**) The contents of dietary fiber in different flesh color. (**J**) The contents of dietary fiber in species). ‘R’—red-fleshed kiwifruit. ‘G’—green-fleshed kiwifruit. ‘Y’—yellow-fleshed kiwifruit. ‘A’—*A. chinensis*. ‘B’—*A. chinensis* var. *deliciosa*. ‘C’—*A. eriantha*. ‘D’—*A. eriantha* × *A. chinensis*. Values are means ± SD of three individual biological reproductions. Values are means ± SD of three individual biological reproductions. Different lowercase letters indicated statistically significant differences (Duncan’s multiple range test, *p* < 0.05) among different kiwifruit cultivars.

The results show that there were significant differences in the energy content, protein content, lipid content, carbohydrates content and dietary fiber content in the 14 kiwifruit varieties. Energy is needed to support daily tasks, and the right amount of energy can help people stay healthy. The highest energy content was found in ‘Hongyang’, with 304.67 KJ/100 g, and which was 30% higher than that of ‘Jinmei’, with the lowest energy content. Kiwifruit samples were classified on the basis of flesh color and species. For color of the flesh, as seen in Figure 2A, the energy content of yellow-fleshed kiwifruits was significantly lower than that of other kiwifruits, and the highest energy was detected in red-fleshed kiwifruits. On the other hand, depending on the species classification, that of *A. chinensis* var. *deliciosa* was significantly higher than *A. eriantha* × *A. chinensis*, as seen in Figure 2B. The energy content of *A. eriantha* × *A. chinensis* was the lowest compared to the other kiwifruit species. In a previous study, the mean values of the energy content in *A. chinensis* and *A. chinensis* var. *deliciosa* were 251.00 KJ/100 g and 255.00 KJ/100 g [20], respectively. In this study, the energy content was higher than in the previous study. It might be that this study had rich samples, which involved six samples of *A. chinensis* and three samples of *A. chinensis* var. *deliciosa*. In addition, the average energy content was 419.14 KJ/100 g in citrus [23], which was far higher than that of kiwifruit.

Carbohydrates are the primary component and source of power for an individual’s existence, and they are included in the composition of energy. The carbohydrate content ranged from 9.07 g/100 g (G6, ‘Huate’) to 14.27 g/100 g (R1, ‘Hongyang’). Interestingly, the highest contents of energy and carbohydrates were both found in ‘Hongyang’. As shown in Figure 2C, the carbohydrates in red-fleshed kiwifruits were statistically higher than those in yellow-fleshed kiwifruits. Depending on the species category, there was no significant difference among the four species in carbohydrates content, as shown in Figure 2D. In the previous study, the average carbohydrate contents of ‘Hort16A’ (*A. chinensis*) and ‘Hayward’ (*A. chinensis* var. *deliciosa*) were 14.23 g/100 g and 14.66 g/100 g [20], respectively. However, in this study, the carbohydrate contents were about 1.4 times lower than those in the previous study. The reason for this might be due to different growing sites and climates in New Zealand and China. Moreover, the average carbohydrate contents were 94.07 g/100 g in apples [22] and 57.55 g/100 g in citrus [23], which was far higher than in this study.

Proteins are vital for human life activities, also helping tissues to build and expand. The highest protein content was 0.87 g/100 g in green-fleshed ‘MHYX’. The mean values of protein content from *A. chinensis* and *A. chinensis* var. *deliciosa* were 1.23 g/100 g and 1.14 g/100 g, respectively, in a previous study [20]. In this study, the results were similar to those for carbohydrate content, which indicated that our results were reliable. In the analyses based on the flesh color of kiwifruits, there are no significant differences between the three types, as shown in Figure 2E. As seen in Figure 2F, based primarily on the different species, the protein contents of *A. chinensis* and *A. chinensis* var. *deliciosa,* as initial commercial varieties, were higher than those of other species. 

Lipids are a vital component of the human body, and the lipid content in the study ranged from 0.59 g/100 g (R2, ‘Donghong’) to 1.01 g/100 g (G5, ‘MHYX’); the lipid content of green-fleshed kiwifruits (0.82 ± 0.14 g/100 g) was significantly higher than that of red-fleshed (0.64 ± 0.06 g/100 g) and yellow-fleshed kiwifruits (0.69 ± 0.08 g/100 g), as shown in Figure 2G. Based on different species, the lipid content of *A. eriantha* was statistical higher than that of other species (Figure 2H). However, the results for *A. chinensis* and *A. eriantha* × *A. chinensis* were similar. Therefore, the hybrid of *A. eriantha* × *A. chinensis* was similar to paternal parents (*A. chinensis*) in this study.

Dietary fiber is an ingredient with a low calorie content that supports normal intestine function. On the one hand, the dietary fiber content of green-fleshed kiwifruits was significantly higher than that of red-fleshed and yellow-fleshed kiwifruits, which was similar to lipid content (Figure 2I). On the other hand, the dietary fiber content of *A. chinensis* was significantly lower than that of *A. chinensis* var. *deliciosa* and *A. eriantha* (Figure 2J). The highest content of dietary fiber was 3.19 g/100 g in ‘Hayward’ in this study, and this was similar to a previous study, in which the dietary fiber content was 3.00 g/100 g in ‘Hayward’ [20]. In addition, the dietary fiber content of ‘Hort16A’ in this study was 1.5 times greater than that of ‘Hort16A’ in this previous study. The reason for this might be due to differences in climate, temperature and geography. Moreover, the average content of dietary fiber is 2.62 g/100 g in kiwifruit. The average dietary fiber contents were 0.98 g/100 g in dragon fruits [21], 2.28 g/100 g in apples [22] and 1.46 g/100 g in tomato fruits [30]. The dietary fiber content in kiwifruit was higher than that in dragon fruit, apple and tomato. Therefore, it suggested kiwifruit has high dietary fiber content.

To sum up, the 14 kiwifruit varieties in this study had the characteristics of low energy and carbohydrates and high dietary fiber and are very suitable for people with “three highs” and poor stomachs.

### 3.4. Total Sugar Content, Sucrose, Glucose and Fructose Content

**Figure 3:**
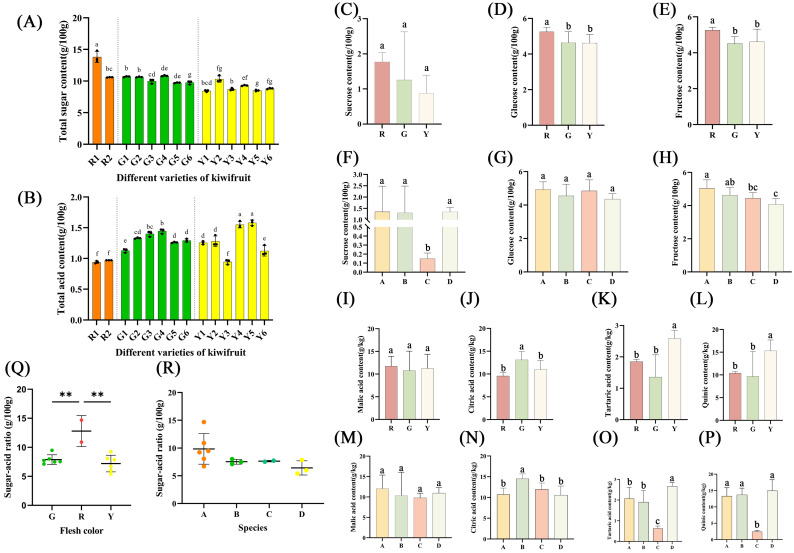
The results of total sugar content, total acid content, sucrose content, glucose content, fructose content, malic acid content, citric acid content, tartaric acid content and quinic acid content and sugar:acid ratio. (**A**) Total sugar content. (**B**) Total acid content. (**C**) Sucrose content in different flesh color. (**D**) Glucose content in different flesh color. (**E**) Fructose content in different flesh color. (**F**) Sucrose content in species. (**G**) Glucose content in species. (**H**) Fructose content in species. (**I**) Malic acid content in different flesh color. (**J**) Citric acid content in different flesh color. (**K**) Tartaric acid content in different flesh color. (**L**) Quinic acid content in different flesh color. (**M**) Malic acid content in species. (**N**) Citric acid content in species. (**O**) Tartaric acid content in species. (**P**) Quinic acid content in species. (**Q**) Sugar:acid ratio in different flesh color. (**R**) Sugar:acid ratio in species. ‘G’—green-fleshed kiwifruit. ‘R’—red-fleshed kiwifruit. ‘Y’—yellow-fleshed kiwifruit. ‘A’—*A. chinensis*. ‘B’—*A. chinensis* var. *deliciosa*. ‘C’—A. eriantha. ‘D’—*A. eriantha* × *A. chinensis*. Values are means ± SD of three individual biological reproductions. Different lowercase letters indicated statistically significant differences (Duncan’s multiple range test, *p* < 0.05) among different kiwifruit cultivars. Statistical significance: ‘**’ *p* < 0.01.

As shown in Figure 3A, the results show that the total sugar content in the samples of different varieties varied significantly. The total sugar content ranged from 8.45 g/100 g (Y1, ‘Hort16A’) to 13.81 g/100 g (R1, ‘Hongyang’), with an average of 10.01 g/100 g, and there were significant differences in the content of soluble sugars in the samples from different varieties. There were no significant differences in sucrose between the groups based on flesh color analysis in Figure 3C. As shown in Figure 3D,E, in the glucose and fructose contents, the red-fleshed kiwifruits greatly outperformed the green-fleshed varieties in terms of flesh color, while the yellow-fleshed varieties did not dramatically distinguish between varieties. However, based on species, the sucrose and fructose contents were significantly different between *A. chinensis* and *A. eriantha*, and the former was higher than the latter in Figure 3F,H. Furthermore, fructose is the sweetest sugar found in nature, and the sweetness of fructose is substantially higher than those of sucrose and glucose. Since fructose does not react with insulin in the human body, it is frequently included in the diets of people with diabetes. The top four varieties with highest fructose content were ‘Hort16A’, ‘G3’, ‘Hongyang’ and ‘Donghong’, and the fructose accounted for approximately 49.82%, 49.23%, 43.86% and 41.68% of soluble sugar in these samples, respectively. Notably, in Figure 3G, the glucose contents of *A. chinensis* strongly outperformed those of *A. eriantha* × *A. chinensis*.

In a previous study, the mean value of the total sugar content in ‘Cuixiang’, ‘Hayward’, ‘Hongyang’, ‘Jintao’, ‘Jinyan’ and ‘Xuxiang’ was 9.26 g/100 g, which was lower than in this study [31]. This might be because fewer samples were used for examination in the earlier studies. In addition, in this study, the average soluble sugar content of ‘Xuxiang’ (10.76 g/100 g), ‘Donghong’ (12.50 g/100 g) and ‘Hongyang’ (12.06 g/100 g) was higher than that in the previous results, where ‘Xuxiang’ was 8.08 g/100 g, ‘Donghong A’ was 10.30 g/100 g, ‘Donghong B’ was 8.91 g/100 g and ‘Hongyang’ was 12.06 g/100 g [32]. The reason for this may be the variation in kiwifruit sugar accumulation in different varieties and different locations.

### 3.5. Total Acid Content, Malic Acid Content, Citric Acid Content, Tartaric Acid Content and Quinic Acid Content

The results show that the total acid content in the samples from different varieties differed significantly, as shown in Figure 3B. The total acid content ranged from 0.94 g/100 g (R1, ‘Hongyang’) to 1.58 g/100 g (Y5, ‘Jinmei’). There were significant differences in the content of organic acids in different species and flesh color groups. On the one hand, the green-fleshed kiwifruit was significantly different from the yellow-fleshed kiwifruit in citric acid, tartaric acid and quinic acid content, whereas there was no significantly differences in malic acid content between green-fleshed and yellow-fleshed kiwifruits (Figure 3I–L). On the other hand, as shown in Figure 3M, in the malic acid content, there were no significant differences among the four species groups. In addition, *A. eriantha* had a significantly lower tartaric acid content and quinic acid content than the other three species groups, as shown in Figure 3O,P.

Some studies showed that the citric acid content and quinic acid content ranged from 4.60 g/kg to 12.90 g/kg and from 5.40 g/kg to 13.54 g/kg [20], respectively, which was consistent with the results of this study, and the tartaric acid content ranged from 3.31 g/kg to 11.79 g/kg [33], which was higher than the experimental results in this study.

### 3.6. Sugar: Acid Ratio

In this study, we classified different kiwifruit samples according to their flesh color and species to analyze the sugar:acid ratio (Figure 3Q,R). The results showed that there were significant differences in the sugar:acid ratio in different varieties. The sugar:acid ratio ranged from 5.40 (Y5, ‘Jinmei’) to 14.67 (R1, ‘Hongyang’). The two highest sugar:acid ratios were detected in ‘Hongyang’ and ‘Donghong’ (10.90), which may account for their widespread acceptability and preferability among consumers worldwide. The content of total sugar in green-fleshed kiwifruits, with an average of 10.27, was similar to that in ‘Donghong’; however, that of total acid was also higher than in ‘Donghong’. Hence, the sugar:acid ratio in green-fleshed kiwifruits was lower than in red-fleshed ones, meaning that red-fleshed kiwifruits taste sweeter than green-fleshed ones. Moreover, yellow-fleshed ‘Jintao’ had a low total sugar content but it had a higher sugar:acid ratio, and so it had a better taste. A previous study showed that the sugar:acid ratio in mature ‘Hongyang’ kiwifruits from Shanxi (China) was 10.39 [34], which was lower than that in this study (14.67). The average temperature in Shaanxi is lower than that in Hubei, so it is reasonable to speculate that the cause of this trend might be the effect of temperature and cultivation management.

### 3.7. Vitamin

In different varieties, the VE content, VC content, VB1 content, VB2 content and VB6 content were significantly different in terms of statistical significance. The highest VE content was 2.29 mg/100 g from ‘Jinyuan’ (hybrid), which was about eight times greater than that of ‘Cuiyu’, with the lowest content of 0.28 mg/100 g. ‘MHYX’ had a far higher VC content than other samples. The highest VB1 and VB2 content were both observed in ‘Hongyang’, with contents of 0.04 mg/100 g for both. The VB6 content ranged from 0.07 mg/100 g (Y2, ‘G3’) to 0.04 mg/100 g (G6, ‘Huate’).

**Figure 4:**
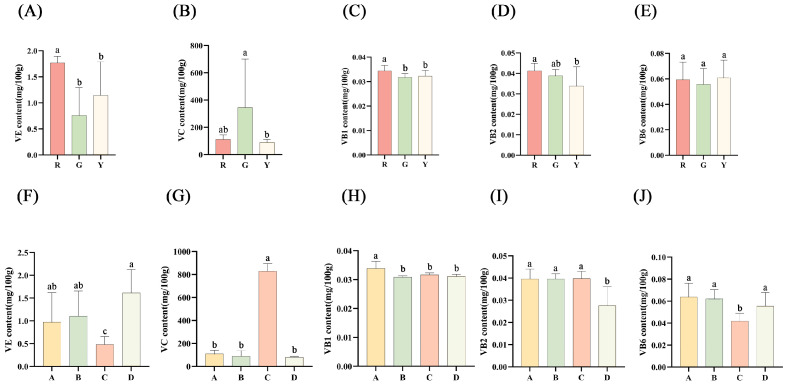
The contents of VE, VC, VB1, VB2 and VB6. ‘G’—green-fleshed kiwifruit. ‘R’—red-fleshed kiwifruit. (**A**) The contents of VE in different flesh color. (**B**) The contents of VC in different flesh color. (**C**) The contents of VB1 in different flesh color. (**D**) The contents of VB2 in different flesh color (**E**) The contents of VB6 in different flesh color. (**F**) The contents of VE in species. (**G**) The contents of VC in species. (**H**) The contents of VB1 in species. (**I**) The contents of VB2 in species. (**J**) The contents of VB6 in species. ‘Y’—yellow-fleshed kiwifruit. ‘A’—*A. chinensis*. ‘B’—*A. chinensis* var. *deliciosa*. ‘C’—*A. eriantha*. ‘D’—*A. eriantha* × *A. chinensis*. Values are means ± SD of three individual biological reproductions. Different lowercase letters indicated statistically significant differences (Duncan’s multiple range test, *p* < 0.05) among different kiwifruit cultivars.

On the one hand, red-fleshed kiwifruits had a statistically significantly higher VE content and VB1 content than green-fleshed kiwifruits, as shown in Figure 4A,C, respectively. However, as seen in Figure 4B, green-fleshed kiwifruits had a far higher VC content than the other flesh colors. *A. eriantha*, which is green-fleshed, is regarded as ‘the king of VC’, which may be the cause of these outcomes. Both VB2 and VB6 content had no trend, with no statistically significant differences in all three flesh colors in Figure 4D,E. On the other hand, although *A. eriantha* had the lowest content of VE, it had the highest VC content, which was about nine times higher than the average for other species (109.22 mg/100 g, 88.34 mg/100 g and 81.39 mg/100 g), as seen in Figure 4F,G. The VB1 content of *A. chinensis* was significantly higher than that in the other three species, and the VB2 content of *A. eriantha* × *A. chinensis* displays the same trend in Figure 4H,I. The content of VB6 in *A. eriantha* was significantly lower than that in other species, and the value of *A. eriantha* × *A. chinensis* was similar to that in parental plants (Figure 4J). A previous study reported that the VC contents of ‘Hongyang’, ‘Cuiyu’ and ‘Jinkui’ in Shaanxi were 100.00 mg/100 g, 88.58 mg/100 g and 111.02 mg/100 g, respectively [34]. In this study, the VC contents of the three varieties from Hubei were about 1.5 times greater than those from Shaanxi. The VB (VB1 + VB2 + VB6) contents of *A. chinensis* and *A. chinensis* var. *deliciosa* in this study were 0.14 mg/100 g and 0.13 mg/100 g, respectively. Compared with one previous study [20], they increased by 8% and 14%, respectively. In summary, the vitamin content of kiwifruits even from the same variety varies in different geographical environments, which was speculated as one of the impact factors in vitamin content.

### 3.8. Aroma Substance

**Figure 5:**
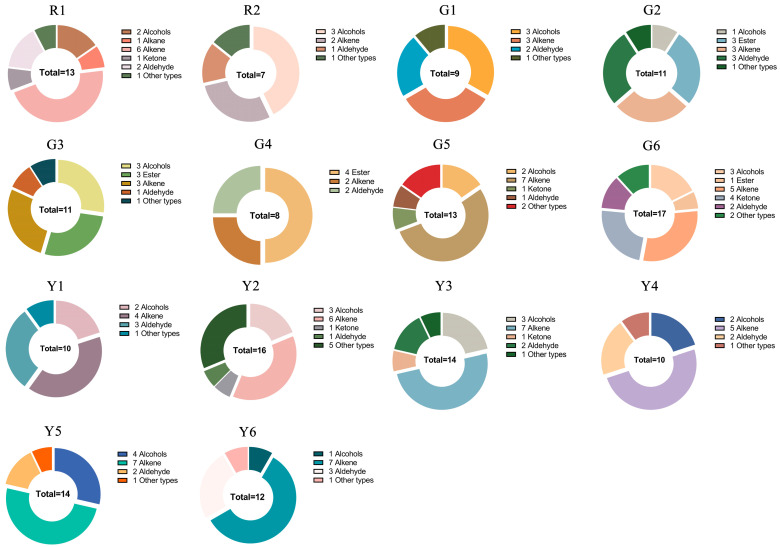
The content of aroma substances. ‘R1’, ‘Hongyang’. ‘R2’, ‘Donghong’. ‘G1’, ‘Cuiyu’. ‘G2’, ‘Xuxiang’. ‘G3’, ‘Hayward’. ‘G4’, ‘Jinkui’. ‘G5’, ‘MHYX’. ‘G6’, ‘Huate’. ‘Y1’, ‘Hort16A’. ‘Y2’, ‘G3’. ‘Y3’, ‘Jintao’. ‘Y4’, ‘Jinyuan’. ‘Y5’, ‘Jinmei’. ‘Y6’, ‘Jinyan’.

Aroma characteristics are among the most important organoleptic characteristics for kiwifruit quality and are one of the crucial concerns of consumers. As shown in Figure 5, in this study, a total of 45 volatile compounds were identified in 14 kiwifruits, including 9 alcohols, 1 alkane, 6 esters, 3 aldehydes, 14 alkenes, 5 ketones and 7 other types. Among them, 17, 16, 14, 14, 13, 13, 12, 11, 11, 10, 10, 9, 8 and 7 volatile components were identified in G6 (‘Huate’), Y2 (‘G3’), Y3 (‘Jintao’), Y5 (‘Jinmei’), R1 (‘Hongyang’), G5 (‘MHYX’), Y6 (‘Jinyan’), G3 (‘Hayward’), G2 (‘Xuxiang’), Y4 (‘Jinyuan’), Y1 (‘16A’), G1 (‘Cuiyu’), G4 (‘Jinkui’) and R2 (‘Donghong’), respectively. Volatile substance is one of the important physical characters in kiwifruit, and different compounds and contents lead to various aromas. In the three flesh color groups, there were 12 common compounds, which were eucalyptol, linalool oxide, terpinene-4-ol, n-hexaldehyde, 2-hexenal, α-pinene, β-pinene, γ-terpinene, D-limonene, α-terpinene, terpinolene and p-cymene. In addition, 1-pentanol, trans-2-hexen-1-ol, n-hexane and styrene were present only in red-fleshed kiwifruits. Therefore, they could be used as a characteristic fragrance for red-fleshed kiwifruits.

It was reported that one of the main aroma compounds was n-hexanaldehyde in ‘Hayward’ (*A. chinensis* var. *deliciosa*) and ‘Hort16A’ (*A. chinensis*) [35]. According to our study, n-hexanaldehyde was also present as a main compound in *A. eriantha* and *A. eriantha* × *A. chinensis*. A previous study found that two compounds, 1-methoxy-2-methyl-benzene and (E, E)-2,4-heptadienal, were only present in *A. chinensis* var. *deliciosa* [18]. In this study, we found six compounds that were only present in *A. chinensis* var. *deliciosa*. The reasons for this might be that the previous research involved one sample in *A. chinensis* var. *deliciosa*, and that limitations in the plant materials led to that conclusion. Another study identified ethyl butyrate, 2-hexenal and n-hexanaldehyde as the three volatile components likely to determine the aroma of ‘Jinyan’ kiwifruit [36]. In this study, n-hexaldehyde and 2-hexenal were present in ‘Jinyan’ kiwifruit, which could narrow down the specific aroma of ‘Jinyan’ kiwifruit.

### 3.9. Principal Component Analysis (PCA)

As seen in Table 2, the 11 quality indicators of the 14 kiwifruit varieties were subjected to principal component analysis and the Kaiser–Meyer–Olkin score was 0.545, demonstrating that these data were suitable for principal component analysis. Three principal components were obtained. The eigenvalues were greater than PCA 1. The total contribution was 79.98%, which represented most of the information of all indicators, in 14 kiwifruit varieties, and can be used as a comprehensive indicator for kiwifruit evaluation.

In order to better explain the relationship between the quality indicators and the principal component factors, we rotated the extracted principal component factors, the magnitude of load reflected the importance of each variable in the principal component, and the analysis results are shown in Table 3. The first principal component was a combination of chlorophyll, carotenoids, flavonoids, lipid, organic acids, VC and pectin, which reflected the color characteristics and nutritional qualities of kiwifruits. The second principal component, including energy, dietary fiber and soluble sugars, reflected the nutritional quality and flavor of kiwifruits. The third component contained protein, which mainly reflected the rate of the fruit’s catalytic metabolism. The results of the calculation of the scores and the overall ranking are shown in Table S1. The top four highest-ranking varieties among the 14 varieties of kiwifruit were ‘Huate’, ‘MHYX’, ‘Jinkui’ and ‘Xuxiang’, respectively. Therefore, we can focus our attention on ‘MHYX’ and ‘Huate’ and vigorously develop their nutritional value to provide processed kiwifruit products for consumers. Furthermore, ‘Jinkui’ and ‘Xuxiang’, as major commercial cultivars, could be vigorously developed to utilize their nutritional value for human health.

## 4. Conclusions

In this study, we estimated pectin, moisture, chlorophyll, carotenoid, flavonoid, energy, carbohydrates, protein, lipid, dietary fiber, soluble sugar, organic acid, vitamin and aroma compound contents according to flesh color and species in kiwifruits. The physical and chemical properties, nutritional properties and aroma varied in different flesh color and species groups. Red-fleshed kiwifruits had the highest sugar:acid ratios and total flavonoid contents, which led to a particularly sweet flavor and a bright red color. However, the energy and carbohydrate contents were much higher in red-flesh kiwifruits, and lipid and dietary fiber contents were lower in red-fleshed kiwifruits than yellow- and green-fleshed kiwifruits. The green- and yellow-fleshed kiwifruits with low energy and carbohydrates were suitable for people with “three highs” and poor stomachs. VE and VB1 contents were high in red-fleshed kiwifruits. Generally, green-fleshed kiwifruits, such as *A. eriantha*, had the highest chlorophyll a content and chlorophyll b content. Interestingly, the total carotenoid content of *A. eriantha* was also the highest in all species, as was the total carotenoid content. The commercial varieties are *A. chinensis* and *A. chinensis* var. *deliciosa,* and the protein contents of fruits belonging to these two species were higher than those in other species. A characteristic aroma for red-fleshed kiwifruits were identified; moreover, the specific aroma of ‘Jinyan’ was narrowed down to being due to n-hexaldehyde and 2-hexenal. The 14 varieties were ranked by principal component analysis, ‘Huate’, ‘MHYX’, ‘Jinkui’ and ‘Xuxiang’ were the top four highest-ranking varieties. Therefore, we can focus our attention on ‘MHYX’ and ‘Huate’ and vigorously make use of their nutritional value to provide processed kiwifruit products. In addition, ‘Jinkui’ and ‘Xuxiang’, as major commercial cultivars, should be further actively promoted. This study facilitates selecting kiwifruits for consumers and provided scientific evidence for kiwifruit processing and utilization.

## Figures and Tables

**Table 1:** Cultivars, species and flesh color of kiwifruits.

Cultivar	Abbr.	Flesh Color	Species
Hongyang	R1	Red	*A.chinensis*
Donghong	R2	Red	*A.chinensis*
Cuiyu	G1	Green	*A.chinensis*
Xuxiang	G2	Green	*A.chinensis* var. *deliciosa*
Hayward	G3	Green	*A.chinensis* var. *deliciosa*
Jinkui	G4	Green	*A.chinensis* var. *deliciosa*
MHYX	G5	Green	*A.eriantha*
Huate	G6	Green	*A.eriantha*
Hort16A	Y1	Yellow	*A.chinensis*
G3	Y2	Yellow	*A.chinensis*
Jintao	Y3	Yellow	*A.chinensis*
Jinyuan	Y4	Yellow	*A.eriantha* × *A.chinensis*
Jinmei	Y5	Yellow	*A.eriantha* × *A.chinensis*
Jinyan	Y6	Yellow	*A.eriantha* × *A.chinensis*

**Table 2:** Eigenvalue and contribution rate of principal components.

PCA	Eigenvalues	Variance Contribution Rate (%)	Cumulative Variance Contribution Rate (%)
1	5.51	50.07	50.07
2	2.07	18.86	68.93
3	1.22	11.05	79.98

**Table 3:** The component matrix after the principal component is rotated on each quality index.

Quality Indicators	PCA		
	1	2	3
Chlorophyll	0.95	0.16	0.01
Carotenoids	0.73	−0.11	0.15
Flavonoids	−0.81	0.44	0.13
Energy	−0.13	0.80	0.37
Protein	−0.22	−0.13	0.80
Lipid	0.87	0.25	0.20
Dietary Fiber	−0.39	−0.58	0.32
Soluble Sugars	−0.56	0.77	0.07
Organic Acids	0.95	0.23	−0.05
VC	0.93	0.30	−0.07
Pectin	0.61	−0.25	0.50

## Data Availability

Data are contained within the article or Supplementary Materials.

## Supplementary Materials

The following supporting information can be downloaded at: https://www.mdpi.com/article/10.3390/foods12010108/s1, Figure S1: Contents of pectin (A,B) and moisture (C,D); Figure S2: Contents of chlorophyll a, chlorophyll b, total carotenoid and total flavonoids; Table S1: Quality prediction and evaluation results of 14 kiwifruits.
