# Supplementary material for: Comparative Study on Physicochemical and Nutritional Qualities of Kiwifruit Varieties
Source: Foods. 2022 Dec 25;12(1):108. doi: 10.3390/foods12010108 (PMC9818353; doi:10.3390/foods12010108)
Supplement: Supplementary file 1 [file foods-12-00108-s001.zip › Figure.pdf]

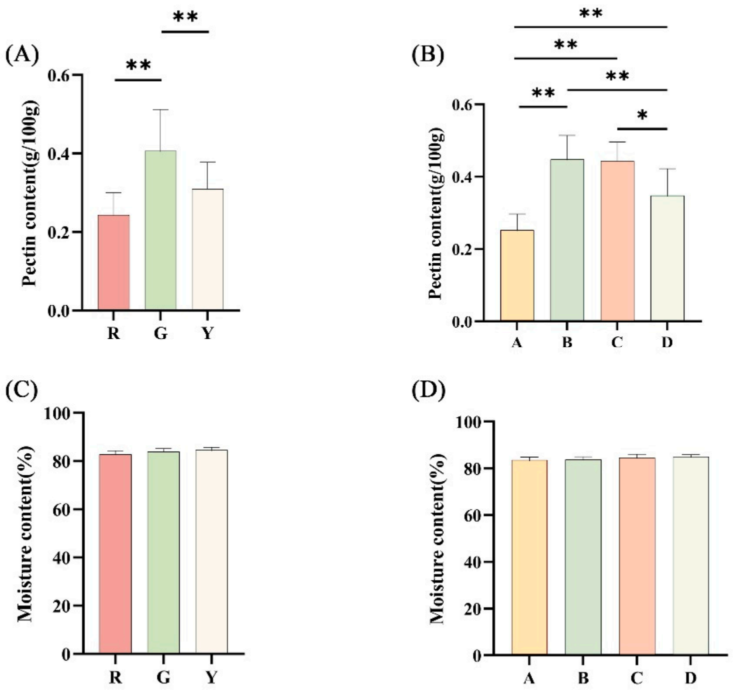

Figure S1. Contents of pectin (A, B) and moisture (C, D). The content in kiwifruits of 3 flesh color (A, C) and 4 species (B, D). 'R' the red-fleshed of kiwifruit. 'G' the green-fleshed of kiwifruit. 'Y' the yellow-fleshed of kiwifruit. 'A' *A.chinensis*. 'B' *A.chinensis* var. *deliciosa*. 'C' *A.eriantha*. 'D' *A.eriantha* × *A.chinensis*. Values are means ± SD of three individual biological reproductions. Statistical significance: '*' $P < 0.05$; '**' $P < 0.01$.

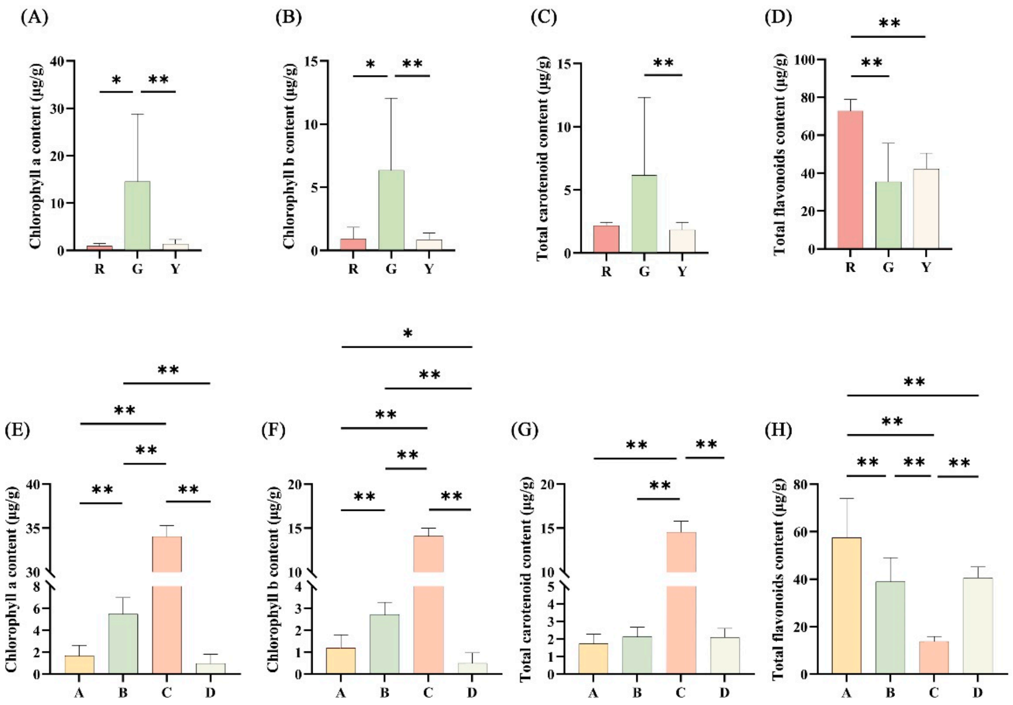

Figure S2. Contents of chlorophyll a, chlorophyll b, total carotenoid and total flavonoids. The content in kiwifruits of 3 flesh color (A-D) and 4 species (E-H). 'R' the red-fleshed of kiwifruit. 'G' the green-fleshed of kiwifruit. 'Y' the yellow-fleshed of kiwifruit. 'A' *A.chinensis*. 'B' *A.chinensis* var. *deliciosa*. 'C' *A.eriantha*. 'D' *A.eriantha* × *A.chinensis*. Values are means ± SD of three individual biological reproductions. Statistical significance: '*' $P < 0.05$; '**' $P < 0.01$.
