# Supplementary material for: Comparative Study on Physicochemical and Nutritional Qualities of Kiwifruit Varieties
Source: Foods. 2022 Dec 25;12(1):108. doi: 10.3390/foods12010108 (PMC9818353; doi:10.3390/foods12010108)
Supplement: Supplementary file 1 [file foods-12-00108-s001.zip › Table.pdf]

Table S1. Quality prediction and evaluation results of 14 kiwifruits.

Varieties	PCA				Rank
	y1	y2	y3	y	
R1	-1.77	2.35	0.49	-39.11	8
R2	-2.08	1.49	-1.24	-89.66	14
G1	-1.54	1.68	0.06	-44.68	9
G2	-0.28	0.19	0.93	-0.29	4
G3	-0.53	-1.90	0.04	-61.88	10
G4	-0.26	0.03	2.70	17.66	3
G5	4.64	1.91	-0.99	257.46	2
G6	5.91	-0.59	0.58	291.15	1
Y1	-1.71	-0.67	0.91	-88.06	12
Y2	-0.31	0.49	-1.20	-19.48	6
Y3	-0.29	-0.94	-0.16	-34.02	7
Y4	-0.08	-0.74	-0.05	-18.43	5
Y5	-0.45	-2.42	-1.20	-81.32	11
Y6	-1.26	-0.89	-0.87	-89.34	13

'R1', 'Hongyang'. 'R2', 'Donghong'. 'G1', 'Cuiyu'. 'G2', 'Xuxiang'. 'G3', 'Hayward'. 'G4', 'Jinkui'. 'G5', 'MHYX'. 'G6', 'Huate'. 'Y1', 'Hort16A'. 'Y2', 'G3'. 'Y3', 'Jintao'. 'Y4', 'Jinyuan'. 'Y5', 'Jinmei'. 'Y6', 'Jinyan'.
